# A TCP Transcription Factor in *Malus halliana*, *MhTCP4*, Positively Regulates Anthocyanins Biosynthesis

**DOI:** 10.3390/ijms23169051

**Published:** 2022-08-12

**Authors:** Jiaxin Meng, Jiao Yin, Han Wang, Houhua Li

**Affiliations:** College of Landscape Architecture and Art, Northwest A&F University, Yangling, Xianyang 712100, China

**Keywords:** anthocyanins, *MhTCP4*, flower color, *Malus halliana*

## Abstract

Anthocyanins belong to a group of flavonoids, which are the most important flower pigments. Clarifying the potential anthocyanins biosynthesis molecular mechanisms could facilitate artificial manipulation of flower pigmentation in plants. In this paper, we screened a differentially expressed gene, *MhTCP4*, from the transcriptome data of *Malus halliana* petals at different development stages and explored its role in anthocyanins biosynthesis. The transcriptome data and qRT-PCR analysis showed that the expression level of *MhTCP4* gradually decreased from the flower color fades. Tissue specific expression analysis showed *MhTCP4* was expressed in the petal, leaf, and fruit of *M. halliana*, and was highly expressed in the scarlet petal. Overexpression of *MhTCP4* promoted anthocyanins accumulation and increased pigments in infected parts of *M.* ‘Snowdrift’ and *M.* ‘Fuji’ fruit peels. In contrast, when endogenous *MhTCP4* was silenced, the anthocyanins accumulation was inhibited and pigments decreased in the infected peels. The qRT-PCR analysis revealed that overexpression or silence of *MhTCP4* caused expression changes of a series of structural genes included in anthocyanins biosynthesis pathway. The yeast two-hybrid assays indicated that MhTCP4 did not interact with MhMYB10. Furthermore, the yeast one-hybrid assays indicated that MhTCP4 did not directly bind to the promoter of *MhMYB10*, but that of the anthocyanins biosynthesis genes, *MhCHI* and *MhF3′H*. Dual luciferase assays further confirmed that MhTCP4 can strongly activate the promoters of *MhCHI* and *MhF3′H* in tobacco. Overall, the results suggest that *MhTCP4* positively regulates anthocyanins biosynthesis by directly activated *MhCHI* and *MhF3′H* in *M. halliana* flowers.

## 1. Introduction

*Malus halliana* is a specie of garden plants with high ornamental value. Flower color is an important ornamental value of plants. With the flower opening, color of *M. halliana* petals gradually fade. The process of flower fade can be divided into three stages, including small bud stage (S1), initial-flowering stage (S2) and late-flowering stage (S3). The color of petals at S1 is scarlet, S2 is pink and S3 is light pink to white. Our previous study has substantiated that the concentration of anthocyanins, cyanidin-3-O-galactoside, in the *M. halliana* flower, decreased significantly during flower fade, which is the essential reason for color fade [1].

At present, the pathway of anthocyanins biosynthesis has been basically identified, and the molecular regulatory network of that is also gradually being improved in *Malus* spp. The structural genes include phenylalanine ammonia lyase (*PAL*), chalcone synthase (*CHS*), chalcone isomerase (*CHI*), flavanone 3-hydroxylase (*F3H*), flavonoid 3′-hydroxylase (*F3′H*), dihydroflavonol 4-reductase (*DFR*), anthocyanin synthase (*ANS*), and UDP-glucose: flavonoid 3-*O*-glu-cosyltransferase (*UFGT*), advance biochemical reactions in anthocyanin biosynthesis [2,3]. In addition, the protein complex MYB-basic-Helix-Loop-Helix (bHLH)-WDR (MBW) has been known as the core regulator in anthocyanins biosynthesis [4,5]. In *Malus*, the three alleles, *MdMYB1*, *MdMYB10*, and *MdMYBA*, were widely reported to play positive roles in anthocyanins biosynthesis under nature or stress [6,7,8]. Moreover, the other transcription factor (TF) families were also reported as anthocyanins biosynthesis regulators in recent years. For example, *PyWRKY26* and *PybHLH3* co-targeted the promoter of *PyMYB114* to enhance anthocyanins biosynthesis and transport in red-skinned pears [9]; and *MdNAC52* positively regulates anthocyanins biosynthesis by interacting with *MdMYB9* and *MdMYB11* promoters [10].

In the recent years, increasing evidence suggests that TCPs play important roles in anthocyanins biosynthesis in plants [11]. TCP (TEOSINTE BRANCHED 1/CYCLOIDEA/PROLIFERATING CELL FACTORS) is a large TF family in higher plants. The TCP proteins play important roles in plant growth and development regulation, plant stress response, and multiple signal transduction pathways [12,13,14]. The typical feature of the TCP family is that it contains a bHLH conserved domain consisting of a longer basic region, two helix and a loop [15]. TCP family is divided into Class I and Class II according to its structural domain characteristics. The differences between them are as follows: one is that Class II has four more amino acids than the TCP domain of Class I, and the other is that the protein of Class II contains a specific R domain rich in 18–20 arginine and glutamate-cysteine-glutamate structure (ECE) [16,17]. Class II can further be divided into two clades, CYC/TB1 clade and CIN clade [15]. Most CYC/TB1-like proteins have R domain, like AtTCP1, AmCYC, and OsTB1, whereas most CIN-like proteins have lost R domain, like AtTCP3, AtTCP4, OsPCF5, and AmCIN [15,18,19,20]. Previous studies have shown that both Class I and Class II TCP proteins are involved in the regulation of anthocyanins biosynthesis. In *A. thaliana*, Class I transcription factor, *AtTCP15*, has been proved to regulate anthocyanins accumulation under high light [21]. And *BrTCP15*, a Class I TCP, inhibits anthocyanin biosynthesis pathway under low light treatments in *Brassicarapa* [22,23]. Class II TCP *AtTCP3* interacting with R2R3-MYB proteins, promotes flavonoid biosynthesis [11]. There is also Class II TCP in *Camellia sinensis* that named *CsTCP3* promoting the anthocyanins accumulation by interacting with CsTT8 and modulating the transactivation activity of the promoters of *CsANS1* and *CsANR1* [24].

In *Malus*, only one Class I TCP gene, *MdTCP46*, was found to interact with MdMYB1, and enhance the binding activity of *MdMYB1* to its target anthocyanins biosynthesis gene promoters at high light intensity [25]. Thus, the identification and function analysis of Class II TCP related to anthocyanins biosynthesis regulation still need explored. In this study, a Class II TCP, named *MhTCP4*, was isolated from *M. halliana* petals. Transient overexpression and silencing assays revealed that *MhTCP4* positively regulates anthocyanins biosynthesis in flower pigmentation. This study provides insights into the transcriptional regulatory mechanisms of TCP family, which increases understanding of the mechanism of pigmentation changes in flowers and therefore will contribute to breed plants with desirable color traits.

## 2. Results

### 2.1. Bioinformatics Analysis of TCP Transcription Factor Family in Malus halliana

**Figure 1:**
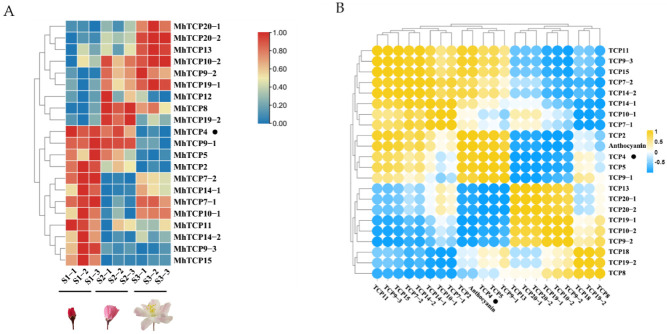
Expression and correlation analysis of *MhTCP*s in *Malus halliana*. (**A**) Heat map of the expression of *MhTCP*s in the petals at three stages. S1, small bud stage; S2, initial-flowering stage; and S3, late-flowering stage. *MhTCP4* is marked by the black dot. (**B**) Heat map representing the correlation between *MhTCP*s expression and the anthocyanin content in the different development stages of petals. Yellow dot represents a positive correlation, and the blue dot represents a negative correlation. *MhTCP4* is marked by the black dot.

In petals of *M*. *halliana* RNA-Seq data, 21 candidate TCPs were screened (Table S1). The expression heat map of FPKM from RNA-seq was shown in Figure 1A. Notably, the expression of *MhTCP4* gradually decreased during flower development, with FPKM values ranging from 203.55 to 517.54, which were higher than that of other *MhTCP*s (Table S1; Figure 1A). In addition, the expression of *MhTCP4* was significantly positively correlated with the anthocyanins content and the correlation coefficient was as high as 0.94 (Figure 1B). The anthocyanins content used in correlation heat map was cyanidin 3-galactoside content in *M. halliana* petals detected by HPLC-DAD, as reported in our previous study [1]. These results proved that *MhTCP4* could play an important role in anthocyanins biosynthesis.

**Figure 2:**
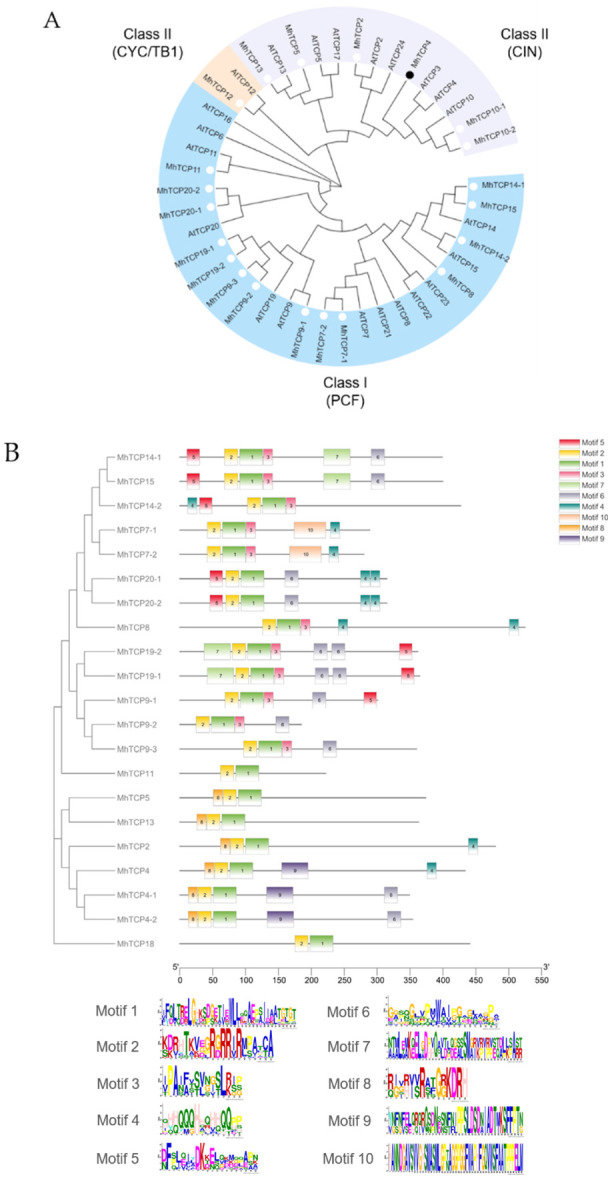

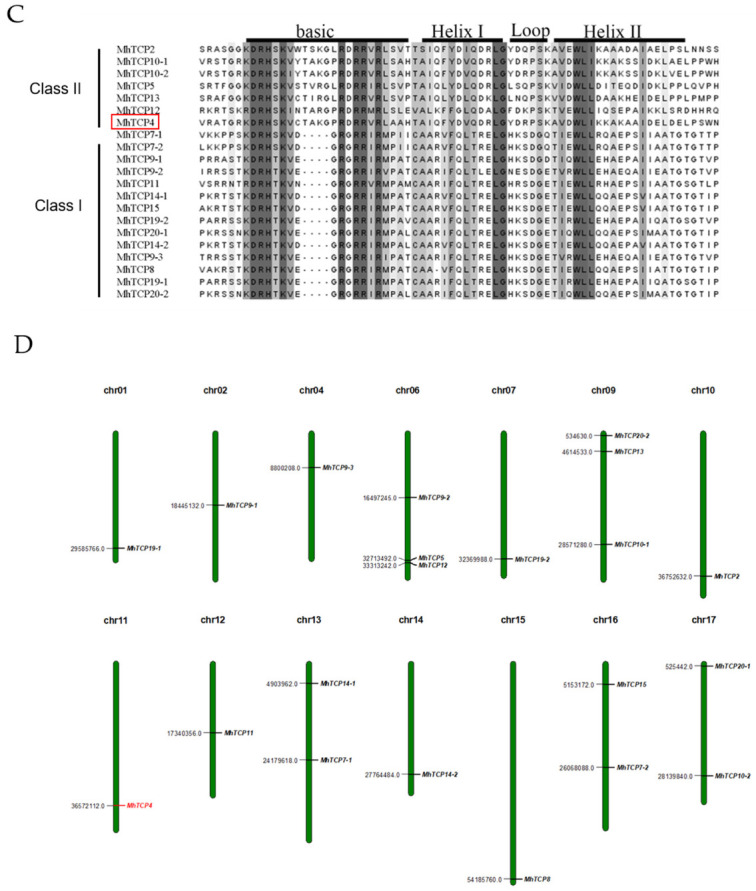
Bioinformatics analysis of *MhTCP* transcription factor family in *Malus halliana*. (**A**) Phylogenetic tree of *MhTCP* transcription factor family. The phylogenetic tree was constructed using the neighbor joining method. *MhTCP4* is marked by the black dot. (**B**) Motif composition of 21 *MhTCP*s. Motifs, numbered 1 to 10, are displayed in the following logos. (**C**) Analysis of conservative amino acid sequences of *MhTCP*s. *MhTCP4* is marked by the red box. (**D**) Chromosomal location of *MhTCP*s. The red gene is *MhTCP4* located in chromosome 11.

Phylogenetic analysis was performed on the 21 candidate *MhTCP*s and 22 *A. thaliana AtTCP*s from the TAIR. The 21 *MhTCP*s were divided into 2 classes. Based on differences in their TCP domains, *MhTCP2*, *MhTCP4*, *MhTCP5*, *MhTCP10-1*, *MhTCP10-2*, and *MhTCP13* were subdivided into CIN clade of Class II, and only *MhTCP18* was subdivided into CYC/TB1 clade of Class II, meanwhile, other *MhTCP*s were divided into Class I (Figure 2A). Through MEME motif analysis, motif 1 and motif 2 were detected in all of 21 TCP protein sequences, which were the basic regions of TCP family (Figure 2B). Compared with class II proteins, the class I proteins were detected four-amino acid deletion in the basic domain (Figure 2C). To determine the chromosomal distribution of the candidate TCP genes, we mapped them into the *Malus* × *domestica* HFTH1 Whole Genome version 1.0 by the MapChart software. The results of chromosomal distribution of the candidate TCPs showed that 21 TCP genes were randomly distributed among 14 chromosomes, in which, Chr06 had three members; Chr10, Chr13, Chr16, and Chr17 contained two genes; and Chr01, Chr02, Chr03, Chr07, Chr10, Chr11, Chr12, Chr14 and Chr15 only contained one gene, respectively (Figure 2D).

### 2.2. Sequence and Phylogenetic Analysis of MhTCP4

**Figure 3:**
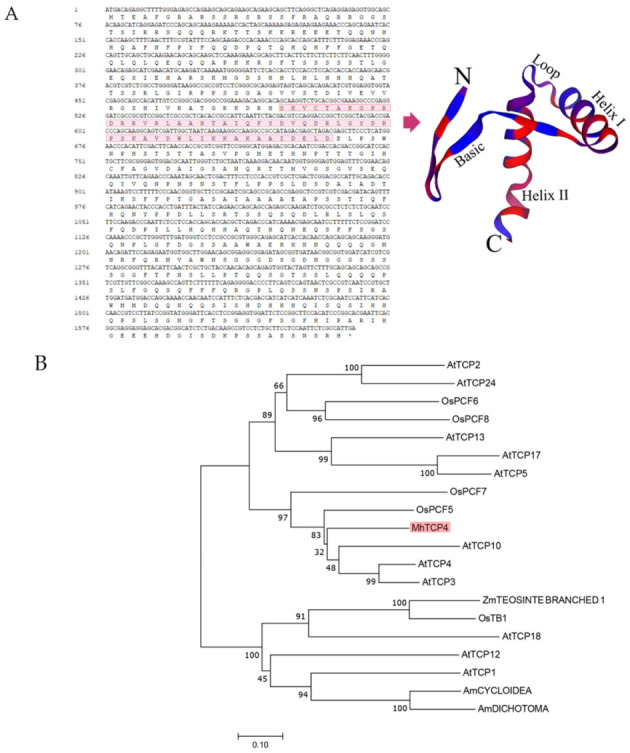

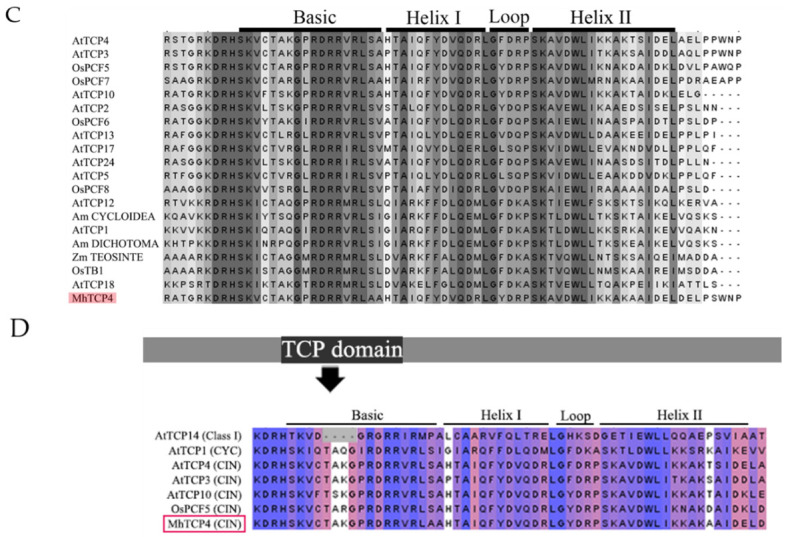
Sequence analysis and phylogenetic analysis of MhTCP4 protein. (**A**) The CDS and amino acid sequence of MhTCP4, and its three-dimensional structural model of protein with conserved domain. Inside the red box is the bHLH domain. (**B**) Phylogenetic analysis of *MhTCP4* and its orthologous genes. The tree was constructed by neighbor-joining method. Branch numbers represent percentage of bootstrap values in 1000 sampling replicates and scale indicates branch lengths. (**C**) Analysis of conservative amino acid sequences of MhTCP4 and its orthologous proteins. A total of 20 proteins were Blast, based on UniProtKB/Swiss-Prot database. The species, gene names, and GeneBank accession numbers are as follows: *Arabidopsis thaliana* (AtTCP1, Q9FYG7.1), *Arabidopsis thaliana* (AtTCP2, Q93V43.1), *Arabidopsis thaliana* (AtTCP3, Q9MAH8.1), *Arabidopsis thaliana* (AtTCP4, Q8LPR5.1), *Arabidopsis thaliana* (AtTCP5, Q9FME3.1), *Arabidopsis thaliana* (AtTCP10, O82277.1), *Arabidopsis thaliana* (AtTCP12, A0AQW4.1), Arabidopsis thaliana (AtTCP13, Q9S7W5.1), *Arabidopsis thaliana* (AtTCP17, Q9LEZ9.1), *Arabidopsis thaliana* (AtTCP18, A1YKT1.1), *Arabidopsis thaliana* (AtTCP24, Q9C758.1), *Oryza sativa* Indica Group (OsPCF5, A2WM14.1), *Oryza sativa* Indica Group (OsPCF7, Q8LT05.2), *Oryza sativa* Indica Group (OsPCF6, A2XMN1.1), *Oryza sativa* Indica Group (OsPCF8, Q2QM59.1), *Oryza sativa* Indica Group (OsTB1, Q8LN68.1), *Zea mays* (ZmTEOSINTE BRANCHED 1, Q93WI2.2), *Antirrhinum majus* (AmDICHOTOMA, Q9SNW8.1), *Antirrhinum majus* (AmCYCLOIDEA, O49250.1). (**D**) MhTCP4 was classified by analyzing the conserved amino acid sequence.

In order to explore the role of *MhTCP4* in anthocyanins biosynthesis, the coding sequence of *MhTCP4* from the petals of *M. halliana* in S1 stage was isolated. Sequence analysis showed that *MhTCP4* gene encodes 546 amino acids. Secondary structure analysis of MhTCP4 protein showed that the conserved domain is mainly maintained by a long basic region, two α-helix and a β-turn containing 5 amino acids (Figure 3A). Then 19 orthologous proteins of MhTCP4 were identified by Blastp, including 11 TCPs in *A. thaliana*, 5 TCPs in rice, 2 TCPs in *Anthirrinum majus* and 1 TCP in maize. Subsequently, phylogenetic analysis of the 21 proteins showed that MhTCP4 was most closely related to OsPCF5, AtTCP3, AtTCP4, and AtTCP10 (Figure 3B). The amino acid sequence alignment of 20 homologous genes, including MhTCP4, shows that the TCP conserved region of these genes is a bHLH domain composed of about 60 amino acid residues (Figure 3C). Referring to the report of Jessica et al. [16], further amino acid sequence analysis confirmed that MhTCP4, AtTCP3, AtTCP4, AtTCP10, and OsPCF5 all belong to CIN clade (Figure 3D). These results suggested that *MhTCP4* may have similar functions of these genes in CIN clade.

### 2.3. Analysis of Tissue Specific Expression of MhTCP4 in Malus halliana

**Figure 4:**
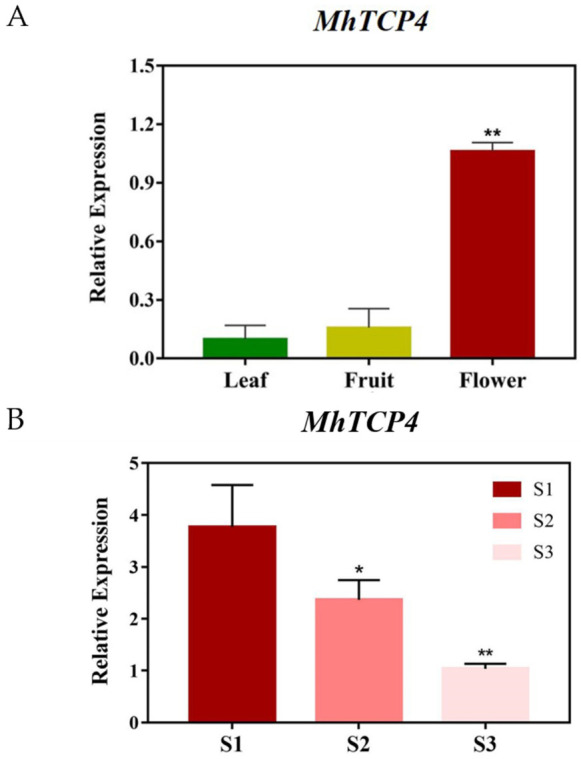
*MhTCP4* tissue specific expression analysis and spatio-temporal differential expression analysis in petals of *Malus halliana.* (**A**) The tissue-specific expression of MhTCP4 was analyzed. The left *y*-axis denotes the RNA relative expression obtained by qRT-PCR. (**B**) Spatio-temporal differential expression of MhTCP4 in the petals of *Malus halliana*. S1, S2 and S3 represent the three different stages of the petals of *Malus halliana*. Error bars represent the SEs of three biological replicates and three technical replicates. * *p* < 0.05, ** *p* < 0.01 using the *t*-test.

The expression pattern of *MhTCP4* in different tissues of *M. halliana* was detected via qRT-PCR. The results showed that *MhTCP4* gene was expressed in leaf, fruit, and petal, but the expression level in petal (S2) was significantly higher than that in fruit or leaf (Figure 4A). In addition, the qRT-PCR results showed that the expression of *MhTCP4* gene gradually reduced from S1 to S3 stage in the petal of *M. halliana* (Figure 4B).

### 2.4. Overexpression of MhTCP4 Promoted Anthocyanins Biosynthesis in Malus spp.

**Figure 5:**
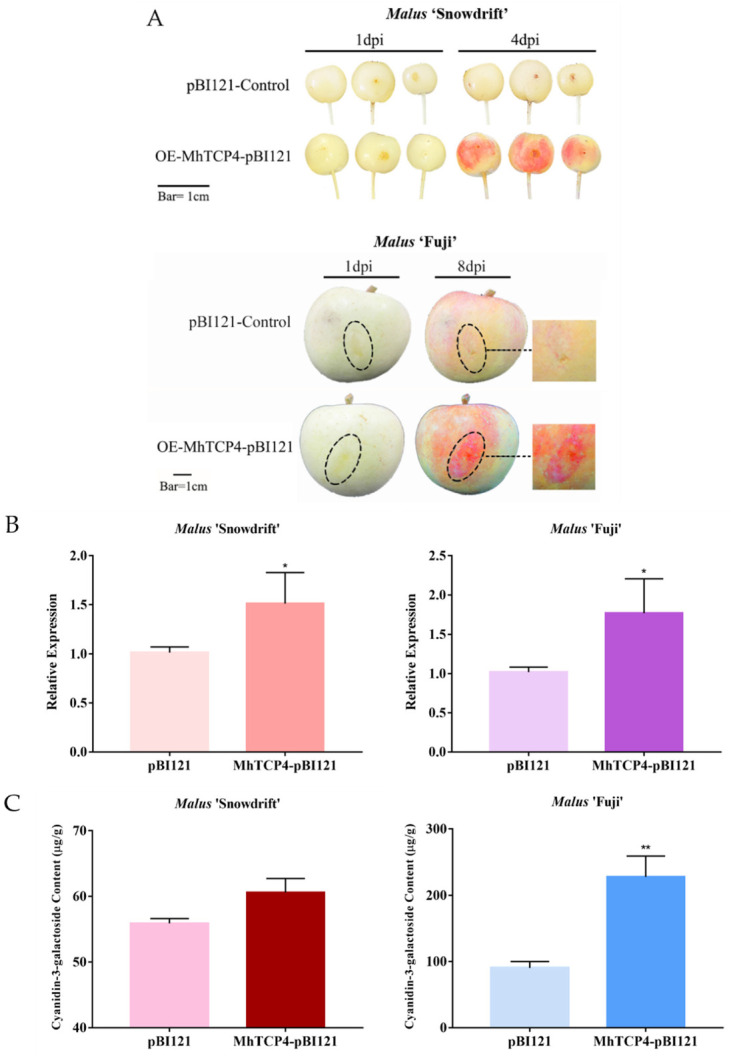

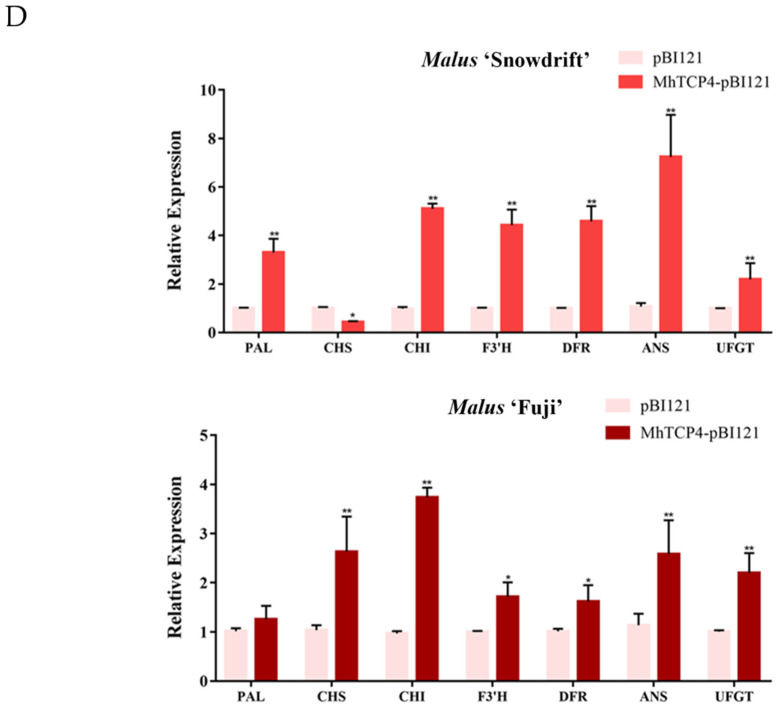
Transient overexpression of *MhTCP4* in fruit peels of *Malus* ‘Snowdrift’ and *Malus* ‘Fuji’. (**A**) Fruit peel coloration around injection sites. MhTCP4-pBI121 were used for overexpression with the pBI121 vector. Empty vectors were the control. (**B**) The cyanidin 3-galactoside content of transgenic and control fruit peels. (**C**) The expression levels of *MhTCP4* in *MhTCP4*-overexpression and control fruit peels. (**D**) The expression levels of anthocyanins biosynthesis genes in *MhTCP4*-overexpression and control fruit peels. Error bars represent the SEs of three biological replicates and three technical replicates. * *p* < 0.05, ** *p* < 0.01 using the *t*-test.

The obvious red pigmentation was observed in fruit peels of *M*. ‘Snowdrift’ and *M.* ‘Fuji’ infected by the bacterial liquid containing recombinant plasmid, respectively. In contrast, no significant phenotypic changes were observed in the peels infected by the empty vector (Figure 5A). Subsequently, the expression level of *MhTCP4* in the experimental group and the control group was detected by qRT-PCR. The results showed that the expression level of *MhTCP4* was up-regulated in the experimental group (Figure 5B). Besides, the anthocyanins from the peels of the infected site were extracted and detected by HPLC. The results showed that an anthocyanin, cyanidin 3-galactoside, was detected in fruit peels of *M.* ‘Snowdrift’ and *M.* ‘Fuji’ (Figure 5C). Compared with the control group, the content of cyanidin 3-galactoside in the fruit peels of *M.* ‘Snowdrift’ and *M*. ‘Fuji’ overexpressed *MhTCP4* was increased by 1.2-fold and 2.5-fold, respectively (Figure 5C). From this point of view, the overexpression of *MhTCP4* significantly promote the anthocyanins biosynthesis. To further clarify the effects of *MhTCP4* overexpression on the molecular regulation pathway of anthocyanins biosynthesis, qRT-PCR analysis was performed on 7 structural genes including *MhPAL*, *MhCHS*, *MhCHI*, *MhF3′H*, *MhDFR*, *MhANS*, and *MhUFGT*. Compared with the control group, the expression of six structural genes included *MhPAL*, *MhCHI*, *MhF3′H*, *MhDFR*, *MhANS*, and *MhUFGT*, except that of *MhCHS* showed an up-regulation trend, which are consistent with that of MhTCP4 (Figure 5D). The results of indicated that the overexpression of *MhTCP4* caused the up-regulation of the expression of anthocyanins biosynthesis pathway genes. Overall, these results suggested that the *MhTCP4* gene overexpression promotes anthocyanins biosynthesis.

### 2.5. MhTCP4 Silencing Inhibits the Anthocyanins Biosynthesis in Malus spp.

**Figure 6:**
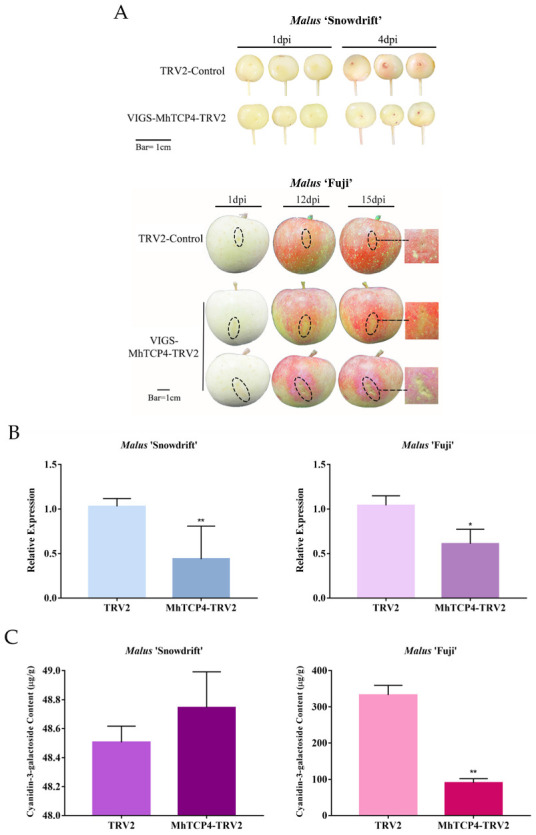

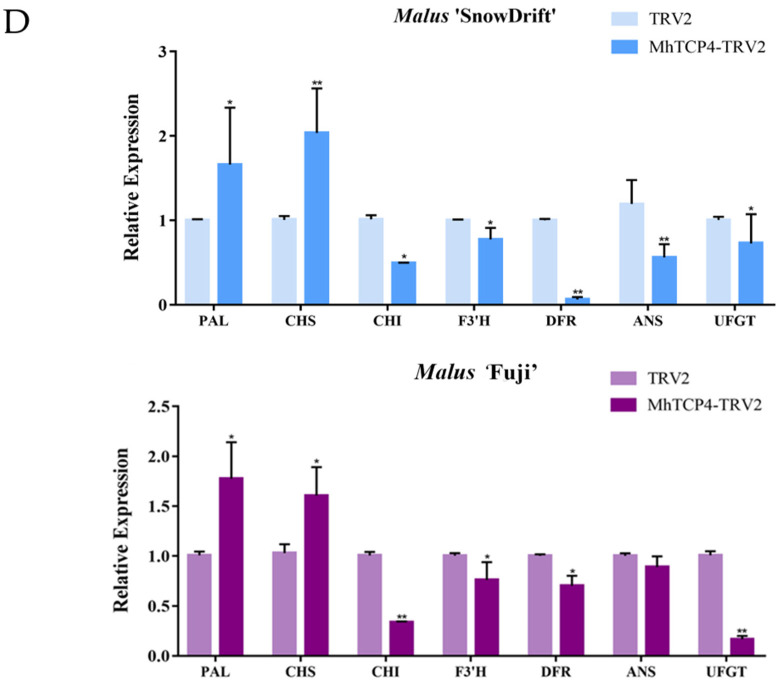
Transient silence of *MhTCP4* in fruit peels of *Malus* ‘Snowdrift’ and *Malus* ‘Fuji’. (**A**) Fruit peel coloration around injection sites. MhTCP4-pTRV2 were used for overexpression with the pTRV2 vector. Empty vectors were the control. (**B**) The cyanidin 3-galactoside content of transgenic and control fruit peels. (**C**) The expression levels of *MhTCP4* in *MhTCP4*-silence and control fruit peels. (**D**) The expression levels of anthocyanins biosynthesis genes in *MhTCP4*-silence and control fruit peels. Error bars represent the SEs of three biological replicates and three technical replicates. * *p* < 0.05, ** *p* < 0.01 using the *t*-test.

Compared with the control group, the red pigmentation accumulation was not observed in fruit peels of *M*. ‘Snowdrift’ where MhTCP4 was silenced. In fruit peels of *M*. ‘Fuji’, a large amount of red pigmentation was accumulated after infection in control group, but the infected parts of ‘peels infected with MhTCP4-TRV2 were remained yellow (Figure 6A). In addition, obvious red pigmentation was observed on the non-infected parts (Figure 6A). Then we performed qRT-PCR detection on the peels of the infected sites in the experimental group and the control group, and the results showed that *MhTCP4* gene in the experimental group was significantly down-regulated both in the infected parts in *M.* ‘Snowdrift’ and *M.* ‘Fuji’ (Figure 6B). The HPLC results showed that the content of cyanidin 3-galactoside in the infected parts of *M.* ‘Snowdrift’ peels and *M.* ‘Fuji’ peels in the experimental group was about 0.8-fold and 0.2-fold of that in the control group, respectively (Figure 6C). And it is speculated that endogenous silencing of *MhTCP4* inhibits the anthocyanins biosynthesis in peels. In *MhTCP4* silencing peels, the expression of structure genes showed a down-regulation trend, which was consistent with that of *MhTCP4* (Figure 6D). Overall, these results suggested that the *MhTCP4* gene endogenous silencing inhibits anthocyanin biosynthesis.

### 2.6. MhTCP4 Not Interacts with MhMYB10

**Figure 7:**
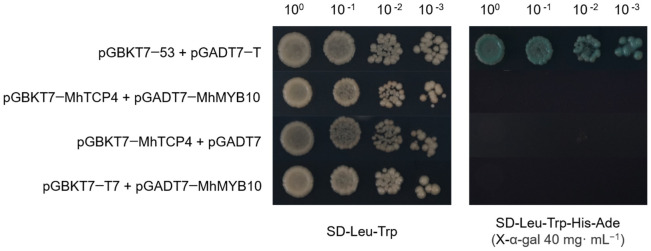
Yeast two-hybrid between MhTCP4 and MhMYB10. The CDS of MdTCP4 and MhMYB10 were fused to the pGBKT7 and pGADT7 vectors, respectively. The pGBKT7-53 + pGADT7-T vectors were used as positive control.

Our previous study substantiated *MhMYB10* (HF36879) positively regulate anthocyanins biosynthesis in *M. halliana* [1]. It would be interesting to analyze whether MhTCP4 is capable of interacting with MhMYB10.As shown in Figure 7, all of these yeast co-transformants could grow normally on SD/-Trp-Leu. However, only the positive control (pGBKT-53 + pGADT7-T) yeast strain grew normally and turned blue on SD/-Trp-Leu-His-Ade (supplemented with 40 mg mL^−1^ X-α-gal), whereas pGBKT7-MhTCP4 + pGADT7-MhMYB10 and the negative control did not grow in the same medium (Figure 7). These results indicated MhTCP4 did not interact with MhMYB10.

### 2.7. MhTCP4 Binds with and Actives Promoter of the Anthocyanins Biosynthesis Genes, MhCHI and MhF3′H

**Figure 8:**
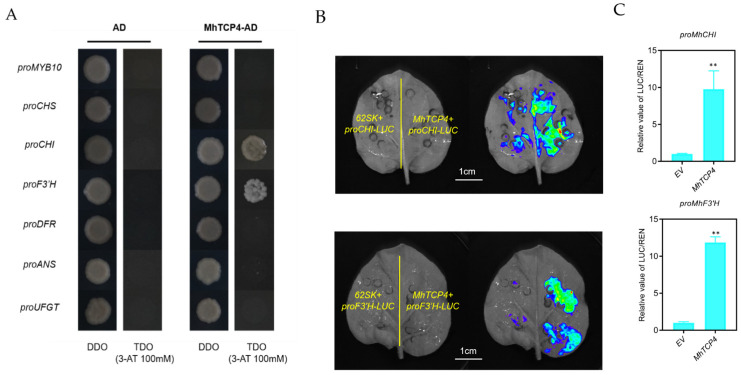
Effect of MhTCP4 on the promoters of anthocyanins-related genes. (**A**) Y1H analysis of the binding of MhTCP4 and the promoters related to anthocyanins biosynthesis. DDO, double-dropout medium (SD/-Leu/-Trp); TDO, triple-dropout medium (SD/-His/-Leu/-Trp). (**B**) Luciferase complementation imaging assays showing that MhTCP4 activates the promotors of *MhCHI* and *MhF3′H*. (**C**) The luminescence intensity of promoters of *MhCHI* and *MhF3′H* increased by MhTCP4. Values are means ± SE of three biological replicates. ** *p* < 0.01 using the *t*-test.

To further determine the functions of *MhTCP4* during anthocyanins biosynthesis, its potential role in binding to the promoter sequences of target genes was examined. In Yeast one-hybrid assays, only proMhCHI-pHIS2 and proMhF3′H-pHIS2 could grow on TDO (SD/-His/-Leu/-Trp) plate with 100 mM 3-AT when the fused pHIS2 vectors were co-expressed with MhTCP4-AD (Figure 8A). Meanwhile, the negative control and other combinations did not grow (Figure 8A). These results suggested that MhTCP4 could not bind to the promoters of the transcription factor, *MhMYB10*, but that of anthocyanins biosynthesis genes, *MhCHI* and *MhF3′H*.

Then, the dual-luciferase assays were performed to detect whether MhTCP4 could activate the promoters of *MhCHI* and *MhF3′H*. As shown in Figure 8B, few LUC luminescence signals were observed from the vectors containing the 62SK and proMhCHI-LUC or 62SK and proMhF3′H-LUC. However, the vectors containing MhTCP4-62SK and proMhCHI-LUC or MhTCP4-62SK and proMhF3′H-LUC vectors produced more stronger LUC luminescence signals (Figure 8B). Compared with the empty vector, the overexpression of *MhTCP4* significantly increased the luminescence signals (Figure 8B). Compared with the empty vector, MhTCP4 increased the *MhCHI* and *MhF3′H* promoter activities by 9.76-fold and 11.86-fold, respectively (Figure 8C). The results were consistent with luminescence signal phenotype. Collectively, these results proved that *MhTCP4* activates *MhCHI* and *MhF3′H* expression by directly binding to their promoters.

## 3. Discussion

It is a common phenomenon that the flower color often gradually fades during development, which are due to the decreased of anthocyanins biosynthesis [26,27,28]. In this study, 21 candidate *TCP*s were screened from transcriptome data in *M. halliana* petals at S1, S2 and S3 stages. Among them, the expression of *MhTCP4* was significantly positively correlated with anthocyanins content of flower development. Through sequence and phylogenetic analysis of *MhTCP4*, 19 orthologous proteins of MhTCP4 were identified. These orthologous proteins are reported to play important roles in flavonoid biosynthesis, leaf morphogenesis, hypocotyl elongation, petal growth and development, leaf development, cell proliferation, and photoperiod flowering regulation [12,18,20,22,29,30,31]. Among them, we found its amino acid sequence and motifs were more similar with *AtTCP3*, which belong to CIN clades of TCP Class II and reported as flavonoid biosynthesis promotor [11]. On account of the functional conservation of TCPs, we speculated that *MhTCP4* may positively regulate anthocyanins biosynthesis as *AtTCP3*. In order to confirm the function of *MhTCP4*, the transient overexpression and silencing were performed in fruit peels off *M*. spp. The *MhTCP4* overexpression in fruit peels of *M.* ‘Snowdrift’ and *M.* ‘Fuji’ induced cyanidin 3-galactoside accumulation and promoted the expression of structural genes related to anthocyanins biosynthesis. By contrast, anthocyanins content and structural gene expression decreased significantly when *MhTCP4* were silenced in peels. Therefore, these results suggest that *MhTCP4* is a functional TCP TF, which promoting anthocyanins biosynthesis.

Except AtTCP3, we have looked at another gene, *OsPCF5*, which also phylogenetically close to *MhTCP4*. In the previous studies, *OsPCF5* and its homologous genes, including *OsPCF8* and *OsPCF6*, have been reported as important regulators response to low temperature stress [14,32]. Another homologous gene, *OsPCF7*, is an important regulatory gene for rice growth and development, and plays multiple roles in rice plant architecture [33]. These studies on the functions of *OsPCF5* and its homologous genes also provide new insights for *MhTCP4* to regulate other functions besides anthocyanin biosynthesis. For instance, in terms of flower development or response to stress in *M. halliana*, whether *MhTCP4* has a function similar to *OsPCF5* remains to be explored in the future.

The previous study has reported AtTCP3 interacts with R2R3-MYB proteins including PAP1 and PAP2 in *Arabidopsis* [33]. TCP family genes encode proteins sharing the TCP domain, a 59-amino acid bHLH motif [15,26], which allows DNA binding and protein-protein interactions [27,28]. However, in this study, the yeast two-hybrid results showed MhTCP4 did not interact with MhMYB10 by the repeated tests. Similar result occurs in MdTCP3, which is also belonged to TCP Class II in *Malus*. MdTCP3 could not physically interact with MdMYB1, an allele of MYB10 with similar protein motifs [25]. Although the conserved TCP domain provides the possibility to interactions, other parts of the proteins with highly divergent, fast evolving sequences outside the TCP domain of different species are, essential for their functional specificity, thus species differences in function may exist [15]. In addition, combined with yeast one-hybrid assay results, the results suggested that MhTCP4 protein could not interact with MhMYB10 protein or directly bind to the promoter of *MhMYB10* to regulate the anthocyanins biosynthesis.

Meanwhile, we confirmed that MhTCP4 directly binds to the promoters of *MhCHI* and *MhF3′H*, while not binds to *MhCHS*, *MhDFR*, *MhANS*, *MhUFGT* through yeast one-hybrid assays. In *Malus*, *MdTCP46*, a Class I TCP gene, is found to enhance the binding activity of MdMYB1 to the promoters of its target gene including *MdDFR* and *MdUFGT* [25]. In addition, previous studies reported MdMYB10 activates the promoter of *MdDFR* and MdMYBA bounds specifically to *MdANS* promoter region [4,6,7]. These results showed that, compared with *MYB10* and its alleles, *MhTCP4* regulates different anthocyanins biosynthesis genes. This is further evidence that the functional role of *MhTCP4* is completely independent of *MhMYB10*. Thus, we extrapolate that, in *M. halliana* flowers, *MhTCP4* and *MhMYB10* concurrently regulate anthocyanins biosynthesis, but the *MhTCP4* and *MhMYB10* regulates different structural genes, respectively.

We noticed that the expression of *PAL* and *CHS* in *M.* ‘Snowdrift’ and *M.* ‘Fuji’ with transient silence *MhTCP4* did not orderly change following the expression of *MhTCP4*. The same situation was reported in previous studies. For example, on the promotion of strawberry anthocyanin biosynthesis by *FvTCP9*, *FvTCP9* interference led to up-regulation of *PAL* expression [34]. We speculated that there is a negative feedback regulatory mechanism between anthocyanins content and the upstream structural genes like *PAL* and *CHS*, since *MhTCP4* could not directly regulates the expression of *PAL* and *CHS* [35]. The UV-B induction was performed during the transient expression assays in fruit peels [36]. Anthocyanins, key compounds for stress resistance, could not be formed in the peels due to the *MhTCP4* silencing [37,38,39]. The absence of anthocyanins caused negative feedback regulation of upstream structural genes, causing an increase in *PAL* and *CHS* expression. In addition, when overexpressing *MhTCP4*, the *CHS* gene expression was inconsistent in the peels of the two specie fruits. This might be due to the sampling time deviation of these two species, resulting in different transient expression levels of *CHS*. Especially in the case that *MhTCP4* cannot directly activate *CHS*, there is a degree of uncontrollable expression of the *CHS* gene. The regulatory network of the anthocyanin biosynthesis pathway is very complex, and further studies are still needed.

In this study, we functionally characterized the *MhTCP4* in *M. halliana* and proved that *MhTCP4* positively regulates anthocyanins biosynthesis. For the first time, we found that MhTCP4 directly activates the promoters of *MhCHI* and *MhF3′H* without interacting with MhMYB10. The activation of *MhCHI* and *MhF3′H* expressions gradually reduces due to the decreased expression of *MhTCP4*, then the anthocyanins synthetic substrate content is decreases and flower fades. Overall, our findings provide new sight on the mechanisms of anthocyanins biosynthesis and will facilitate artificial manipulation of flower pigmentation in ornamental plants.

## 4. Materials and Methods

### 4.1. Plant Materials and Growth Conditions

*M. halliana* petals, *M.* ‘Snowdrift’ fruits and *M.* ‘Fuji’ fruits were used as research materials, *M. halliana* and *M*. ‘Snowdrift’ all grew in Northwest A&F University, Shaanxi, Yangling, China (34°20′ N, 108°24′ E), *M*. ‘Fuji’ fruits were collected from “Chunhua Tiandi” Orchard in Chunhua County, Shaanxi Province.

Petals of *M. halliana* were collected from three different periods (S1, S2, S3). Due to the fruits of *M. halliana* are not able to turn red, the fruits of *M.* ‘Snowdrift’ and *M.* ‘Fuji’ were used to transient expression experiment. *M.* ‘Snowdrift’ fruits were bagged for 20 days, and *M.* ‘Fuji’ fruits were bagged for 35 days after falling flower until used, respectively. The petals, leaves and peels were immediately frozen in liquid nitrogen, then they were stored at −80 ℃ for further experiments.

### 4.2. RNA Sequencing Data Analysis

Library construction, RNA sequencing (RNA-Seq), RNA assembly, and DEG analysis of *Malus halliana* petals at S1, S2, and S3 stages, each with three biological replicates, were performed and reported in our previous study [1].

### 4.3. Bioinformatics Analysis of TCP Gene Family in Malus halliana

Compared with Genome Database for Rosaceae (GDR, https://www.rosaceae.org/) (accessed on 16 May 2019), the candidate TCP genes were screened from *M. halliana* transcriptome database. The heat map of gene expression based on FPKM was constructed by local software TBtools [40]. The FPKM values of the *MhTCP* TF family genes were taken in the transcriptome, and the total anthocyanins contents of the three development stages (S1, S2, S3) in *M. halliana* were taken, and the local software TBtools was used to draw the correlation analysis heatmap [40].

The TCP protein sequences of *M. halliana* and *A. thaliana* were alignment. The alignment results were imported into MEGA 7, and the neighbor joining (NJ) method was used to construct a phylogenetic tree [41]. The parameter settings were as follows: bootstrap method 1000; P-distance model; partial deletion; cutoff 50. Then, the online software Evolview (www.evolgenius.info/evolview) (accessed on 20 May 2019) was used to beautify the phylogenetic tree [42]. Online software MEME (https://meme-suite.org/meme/)(accessed on 18 June 2019) and local software TBtools was used to identify and visualize the conserved domain motif [40,43]. To determine the chromosomal distribution of the candidate TCP genes, we mapped them into the *Malus* × *domestica* HFTH1 Whole Genome version 1.0 by the MapChart software [44].

The obtained *MhTCP4* gene sequence was analyzed, and the CDS (coding sequence) was translated by DNAMAN software. The translated amino acid sequence was analyzed by SWISS-MODEL online software (https://swissmodel.expasy.org/) (accessed on 15 December 2019) to analyze the three-dimensional protein structure of *MhTCP4* conserved domain [45]. To analyze the potential functionality of *MhTCP4*, the orthologous genes which have been annotated in other species of *MhTCP4* gene were analyzed by Blastp (https://blast.ncbi.nlm.nih.gov/Blast.cgi) (accessed on 21 July 2020).

### 4.4. Extraction of Total RNA and Isolation of the MhTCP4 Genes

Total RNA was extracted from *M*. halliana petals with EZNA Plant RNA Kit (R6827-01, Omega Bio-tek, Norcross, GA, USA) according to the manufacturer’s protocols, after which 1000 ng RNA was used as the template to reverse-transcribe to first-strand cDNA with the TransScript^®^ One-Step gDNA Removal and cDNA Synthesis SuperMix Kit (TransGen, Beijing, China). According to the gene login number HF03916, the coding DNA sequence (CDS) of *MhTCP4* homologous gene (*MdTCP4*) was downloaded from GDR, and a pair of upstream and downstream primers was designed using Primer premier 5.0 software (Table S2). Next, PCR products were attached to pMD-19T vector (TaKaRa, Dalian, China).

### 4.5. Overexpression and Silence Recombinant Plasmid Construction

To overexpress the *MhTCP4* gene, a plant binary expression vector pBI121 that containing the CaMV 35S promoter was choosed. Restriction sites were introduced at both ends of upstream and downstream primers to amplifying CDS of *MhTCP4* gene, and then inserted between the Sac I and Xma I double restriction sites of pBI121 vector to construct the recombinant plasmid 35S: MhTCP4-pBI121 (Table S2).

In order to specifically silence *MhTCP4*, the primers were designed to clone a 355 bp sequence in the 3′untranslated region (3′UTR) of *MhTCP4*, which was used in the subsequent VIGS experiment (Table S2). The recombinant plasmid was constructed using the modified TRV2 virus vector. After analyzing the restriction sites of 3′UTR-MhTCP4 and TRV2 vector, the 3′UTR-MhTCP4 was inserted between the Sma I and BamH I restriction sites of the TRV2 vector to generate gene silencing recombinant plasmid MhTCP4-TRV2.

### 4.6. MhTCP4 Overexpression or Silence in Fruit Peels of Malus ‘Snowdrift’ and Malus ‘Fuji’

The constructed recombinant plasmid was transformed into *Agrobacterium tumefacienes* strain GV3101. For overexpression of *MhTCP4* gene, based on the method of Li et al. [46], the method of infection was adjusted appropriately. The fruits of *M.* ‘Snowdrift’ and *M.* ‘Fuji’ were injected by 1 mL sterile needle removal syringe. Empty vector, pBI121, was used as control. All the treated fruits were placed in the dark at 4 °C for 24 h and then cultured in a 17 °C culture chamber under continuous light for 24 h, supplemented with UV-B (254 nm–315 nm, Philips, 40 W), phenotype of infected fruit was observed 4 or 8 days after infection.

VIGS experiment was adapted from the method of Li et al. [46]. The *A. tumefacienes* containing TRV1 and MhTCP4-TRV2 were mixed in a 1:1 ratio. At the same time, TRV1 and empty TRV2 plasmid were mixed in a 1:1 ratio as the control group. Then the mixed solution was incubated in a shaker at 28 °C for 90 r for 3 h in the dark. The infection treatment and the store conditions of *M.* ‘Snowdrift’ and *M.* ‘Fuji’ fruits are the same as that mentioned above.

### 4.7. qRT-PCR Analysis

Total RNA was extracted from the peels of the infected site and was reverse-transcribed into first-strand cDNA using TransScript^®^ One-Step gDNA Removal and cDNA Synthesis SuperMix Kit (TransGen, Beijing, China) according to the manufacturer’s instructions. The real-time quantitative PCR using 2 × SYBR real-time PCR mixture kit (BioTeKe, Beijing, China) on the StepOnePlus real-time PCR system (Applied Biosystems, Waltham, MA, USA). Three biological replicates with three technical replicates were performed for each genetic test, and the transcript abundance was calculated according to the 2^−ΔΔCt^ method. The primer sequences are shown in Supplementary Table S2.

### 4.8. HPLC Analysis

The extraction of phenolic compounds was described previously [8,47]. The extraction supernatant was absorbed and filtered through a 0.45 µm filter, which was analyzed by HPLC coupled with a diode array detector (DAD) (Shimadzu LC-2030C Liquid Chromatograph, Shimadzu, Kyoto, Japan; Inertsil C-18 column, 5.0 µm particle size, 4.6 mm × 250 mm). HPLC separation was performed using a linear gradient of A (10% formic acid dissolved in water) and B (10% formic acid and 1.36% water in acetonitrile) at 30 °C at a flow rate of 1.0 mL·min^−1^. Phenolic compounds were identified by reference to the UV spectral absorption peak and retention time of known standards, the content of phenolic compounds was calculated using standard calibration curves. Three biological replicates with three technical replicates were measured for each sample.

### 4.9. Yeast Two-Hybrid Assay

The CDS of *MhTCP* were inserted into pGBKT7 vector between Nde I and BamH I, and *MhMYB10* was inserted into pGADT7 vector Nde I and BamH I, respectively. The primers used for the Y2H assays are listed in Supplementary Table S2. The recombinant plasmids were co-transformed into yeast strain Y2H Gold and plated on medium lacking Trp and Leu (SD/-Trp-Leu) at 28 °C. Then the Yeast transformed with the positive control (pGBKT7-53 + pGADT7-T), pGBKT7-MhTCP + pGADT7-MhMYB10 and the negative control (pGBKT7-MhTCP + pGADT7; pGBKT7 + pGADT7-MhMYB10) were transferred to medium lacking Trp, Leu, His, and Ade (SD/-Trp-Leu-His-Ade) with 40 mg·mL^−1^ X-α-Gal for interaction screening.

### 4.10. Yeast One-Hybrid Assay

The promoter fragments of the anthocyanins biosynthesis related gene including MYB10, CHS, CHI, F3′H, DFR, and ANS, were amplified from genomic DNA extracted from *M. halliana* petals were inserted into the reporter vector pHIS2 between EcoR I and Sac I. The CDS of MhTCP4 was inserted into the pGADT7 vector between Nde I and BamH I. The primers used for Y1H assay are listed in Supplementary Table S2. The recombinant plasmids were co-transformed into yeast strain Y187 and plated on screening medium lacking Trp and Leu (SD/-Trp-Leu) at 28 °C. Then screened on selective medium (SD/−Leu/−Trp/−His) containing the optimal concentration of 100 mM 3-amino-1,2,4-triazole (3-AT).

### 4.11. Dual LUC Reporter Assay

To screen for downstream genes of *MhTCP4*, the ORF sequence of *MhTCP4* were inserted into pGreenII 62-SK effector vectors between EcoR I and Kpn I. The promoter fragments from *MhCHI* and *MhF3′H* were inserted into the reporter vector pGreenII 0800-LUC between Sal I and BamH I. The empty vector pGreenII 62-SK was used as a negative control effector. Transformation and infiltration were performed as described in Yang et al. [48]. The luciferase signals were observed by an imaging apparatus (Plant View 100, Guangzhou, China). The transcriptional activity was examined by a luciferase detection Kit (Transgen, Beijing, China). The transcriptional abilities were expressed by the LUC/REN ratio.

## Data Availability

Not applicable.

## Supplementary Materials

The following supporting information can be downloaded at: https://www.mdpi.com/article/10.3390/ijms23169051/s1.
